# Research Strategies in the Study of the Pro-Oxidant Nature of Polyphenol Nutraceuticals

**DOI:** 10.1155/2011/467305

**Published:** 2011-06-26

**Authors:** Harvey Babich, Alyssa G. Schuck, Jeffrey H. Weisburg, Harriet L. Zuckerbraun

**Affiliations:** Department of Biology, Stern College for Women, Yeshiva University, 245 Lexington Avenue, New York, NY 10016, USA

## Abstract

Polyphenols of phytochemicals are thought to exhibit chemopreventive effects against cancer. These plant-derived antioxidant polyphenols have a dual nature, also acting as pro-oxidants, generating reactive oxygen species (ROS), and causing oxidative stress. When studying the overall cytotoxicity of polyphenols, research strategies need to distinguish the cytotoxic component derived from the polyphenol *per se* from that derived from the generated ROS. Such strategies include (a) identifying hallmarks of oxidative damage, such as depletion of intracellular glutathione and lipid peroxidation, (b) classical manipulations, such as polyphenol exposures in the absence and presence of antioxidant enzymes (i.e., catalase and superoxide dismutase) and of antioxidants (e.g., glutathione and *N*-acetylcysteine) and cotreatments with glutathione depleters, and (c) more recent manipulations, such as divalent cobalt and pyruvate to scavenge ROS. Attention also must be directed to the influence of iron and copper ions and to the level of polyphenols, which mediate oxidative stress.

## 1. Introduction

Concerns to reduce the risk of chronic diseases have led to much research to discern those lifestyle choices that potentially lessen developing chronic pathologies, cancer in particular. Epidemiological evidence has shown that the dietary consumption of fruits and vegetables can mediate the risk of developing malignancies. Studies with laboratory animal models and *in vitro* research with cells in culture have confirmed the anticarcinogenic effects of natural phytochemicals. In particular, the polyphenol components of phytochemicals have been identified as anticarcinogens. The most studied polyphenol is (−)-epigallocatechin-3-gallate (EGCG), the major constituent in green tea. Such nonnutritive polyphenol phytochemicals are termed nutraceuticals, and their ready bioavailability makes their consumption as potential cancer chemopreventive agents a meaningful lifestyle choice [1]. 

 Polyphenols, a heterogeneous class of phytochemicals with a wide range of pharmacological properties, are most known for their antioxidant properties and their abilities to act as scavengers of reactive oxygen species (ROS). ROS include hydrogen peroxide (H_2_O_2_), superoxide anion (O_2_^·−^), and hydroxyl radical (OH^·^). ROS are formed as by-products of mitochondrial respiration or by certain oxidases, such as nicotine adenine dinucleotide phosphate (NADPH) oxidase. ROS are involved in many cellular events, including as second messengers in the activation of several signaling pathways leading to the activation of transcription factors, mitogenesis, gene expression, and the induction of apoptosis, or programmed cell death [2–4]. Overproduction of ROS, as indicated by a change in the redox state of the cell, may lead to oxidative damage of proteins, lipids, and DNA. To prevent oxidative stress, neutralization of excessive ROS is accomplished by antioxidant enzymes, including superoxide dismutase (SOD) to detoxify O_2_^·−^ and catalase and glutathione peroxidase to detoxify H_2_O_2_. In addition, the tripeptide, glutathione (*γ*-glutamylcysteinylglycine; GSH), plays a major role in maintaining intracellular redox balance and in alleviating ROS-induced oxidative stress. Synthesized enzymatically by *γ*-glutamylcysteine synthetase and glutathione synthetase, a prime function of GSH is to scavenge ROS and thereby to prevent oxidative damage [5]. Because oxidative stress has been implicated with cancer, as well as with other chronic diseases and pathologies, including atherosclerosis, neurodegenerative diseases, and aging, much research has focused on the antioxidant properties of plant-derived polyphenols. 

 Interestingly, plant-derived antioxidant polyphenols have both pro-oxidative and antioxidative properties, depending on such factors as their metal-reducing potential, chelating behavior, pH, and solubility characteristics [6]. Fukumoto and Mazza [7] noted dual antioxidant and pro-oxidant activities for a variety of plant-derived polyphenols including gallic acid, protocatechuic acid, syringic acid, vanillic acid, ellagic acid, caffeic acid, coumaric acid, chlorogenic acid, ferulic acid, myricetin, quercetin, rutin, kaempferol, (+)-catechin, (−)-epicatechin, delphinidin, and malvidin. The volume of research on the antioxidant properties of polyphenols as related to their biological effects greatly overshadows the lesser number of studies on the biological consequences of the pro-oxidant nature of polyphenols. 

 When interpreting cellular responses to a polyphenol, attention must be focused on the effect evoked by the polyphenol *per se*, as distinct from the effect evoked through its generation of significant levels of ROS. For example, Vittal et al. [8] studied gene expression in Ha-*ras *gene-transformed human bronchial epithelial 21BES cells exposed to 25 *μ*M EGCG in the absence and presence of catalase, 30 U/mL. Their use of DNA microarray analyses allowed for distinction between those genes whose expressions were H_2_O_2_ independent from those whose expressions were H_2_O_2_ dependent (i.e., their expressions were abolished by catalase). Gene expression, studied at varying time intervals over a 48-hour period of exposure, indicated time-dependent expression patterns. Many of the H_2_O_2_-dependent genes were early response or biphasic genes associated with cell cycling, whereas, the H_2_O_2_-independent genes were either intermediate- or late-response genes. The initial cellular response was to H_2_O_2_ and, thereafter, to EGCG. The presence of catalase eliminated H_2_O_2_ induction of apoptotic genes (*TXA2R*, *TNFRSF6*, and *MADD*) and slowed down the rate of apoptosis. However, apoptotic cell death still occurred but after a delay of 12 to 24 hr, indicating that EGCG *per se* induced H_2_O_2_-independent apoptosis.

 The intent of this paper is not to review the molecular biology of the various signaling and transducing pathways ignited upon exposures to polyphenols [2, 9, 10]. Rather the goal is to discuss research strategies, some classical and others novel, to demonstrate oxidative stress as the causative agent of polyphenol-induced biological effects, in particular, antiproliferative and proapoptotic effects to cancer cells. To clarify the molecular mechanism whereby a polyphenol exerts an anticarcinogenic effect, it is important to differentiate between the polyphenol *per se* and its ROS auto-oxidation products.

## 2. Generation of Pro-Oxidants

The pro-oxidant characteristic of polyphenols, as noted by their abilities to generate ROS, has been shown both in cell-free systems and in *in vitro* studies with cells. ROS have been detected in cell culture media and in phosphate buffers amended with polyphenols. Time-dependent generation and concentration-dependent generation of H_2_O_2_ were noted in Dulbeccco's modified Eagle medium (DMEM) amended with green tea, red wine [11], green tea polyphenol extract, black tea polyphenol extract [12], *Ginkgo biloba* extract [13], pomegranate extract [14], apple extract [15], EGCG, epigallocatechin (EGC) [12, 16], epicatechin gallate (ECG) [17], catechin gallate [18], theaflavin, theaflavin-3-monogallate, theaflavin-3′-monogallate, theaflavin-3,3′-digallate (TFdiG) [19, 20], chrysin [21], gallic acid [15, 16, 22], and quercetin [15, 16]. The quantity of H_2_O_2_ generated was dependent upon the specific medium. EGCG, EGC, gallic acid [16], and pomegranate extract [14] generated greater levels of ROS in DMEM, as compared to in RPMI 1640 and McCoy's media. Instability of the polyphenol at alkaline pH, resulting in its auto-oxidation, accounted for the generation of ROS in cell culture media, which most commonly was quantified by the FOX assay. The basic principle of this method is the oxidation of ferrous ions (Fe^2+^) by the pro-oxidant polyphenol to ferric ions (Fe^3+^), which bind with xylenol orange to give a colored complex.

**Figure 1:**
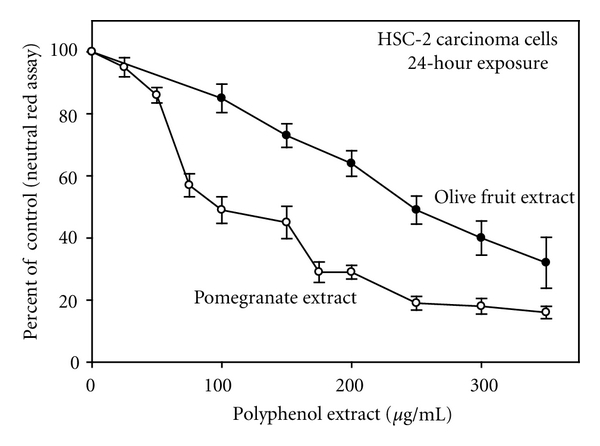
Comparative toxicities of pomegranate extract and olive fruit extract towards proliferation of human oral HSC-2 carcinoma cells as quantified with the neutral red assay, a cell viability assay. Data are presented as the mean percent of control ± S.E.M. Data for the pomegranate study are from Weisburg et al. [14] and for the olive fruit extract from Schuck (unpublished).

 The cytotoxicity of a polyphenol is dependent both on the specific polyphenol *per se *and upon the amount of generated ROS. Gallic acid generated considerably more H_2_O_2_ in DMEM than did quercetin and also exerted stronger antiproliferative activity than quercetin both to human colon Caco-2 cancer cells and to normal rat liver WB-F344 epithelial cells [22]. In DMEM, pomegranate extract generated more H_2_O_2_ than did olive fruit extract (i.e., a 2-hour incubation of 250 *μ*g/mL extract in DMEM yielded 113.9 ± 7 and 51.5 ± 2 *μ*moles/L H_2_O_2_, resp.) and exerted greater growth inhibition to oral HSC-2 carcinoma cells (Figure 1) ([14]; Schuck, unpublished). 

**Figure 2:**
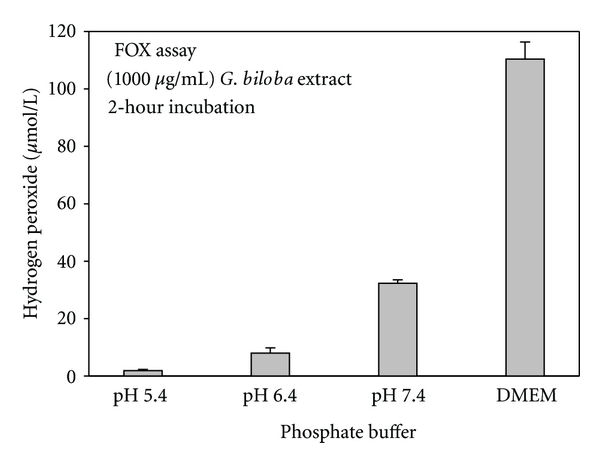
Comparative generation of hydrogen peroxide (H_2_O_2_) in phosphate buffer, maintained at different pH levels, and in cell culture medium supplemented with *Gingko biloba* extract. The Dulbecco's modified Eagle medium (DMEM) in this study was amended 10% Serum Plus, 2% fetal bovine serum, and antimicrobial agents and was the medium in which the cells were exposed to the test agents. H_2_O_2_, generated by the plant extract, was quantified with the FOX assay, after a 2-hour incubation at room temperature. Data, from Babich et al. [13], are presented as the mean percent of H_2_O_2_  ± S.E.M.

 The components of the cell culture medium, including the various inorganic salts, vitamins, and amino acids, contribute to, but are not completely essential for, the generation of ROS by a polyphenol. ROS were noted in polyphenol-amended phosphate buffer although lesser amounts of ROS were detected in phosphate buffer than in cell culture medium [13, 14, 19, 22, 23]. pH is an important factor in moderating the generation of ROS by polyphenols. Generation of H_2_O_2_ occurred in EGCG-amended sodium phosphate buffer at pH 7.8, with lesser amounts at pH 6.8 and no detection at pH 5.8 [23]. Similar findings for the pH-mediated generation of ROS in phosphate buffer were noted with *G. biloba* extract (Figure 2) [13], pomegranate extract [14], and black tea theaflavins [19].

 Another approach compared the intracellular ROS levels in unamended cells and in cells treated with polyphenols. The most common methodology utilizes the diacetate ester of 2′,7′-dichlorodihydrofluorescein (DCHF-DA), a colorless, nonfluorescent, nonpolar molecule that passively diffuses into cells. Within the cell, esterases cleave the two acetates to form DCHF, a nonpermeable, polar molecule. Oxidation of the trapped nonfluorescent DCHF by ROS, principally H_2_O_2_, yields the fluorescent product, 2′,7′-dichlorofluorescein. Fluorescence is quantified with a fluorometer, flow cytometer, microplate spectrophotometer, or a fluorescence microscope [24].

 This methodology identified elevated levels of intracellular ROS in EGCG-treated H661 human lung cancer cells [25], leukemic UF-1 cells, freshly isolated leukemic cells from patients [26], Burkitt lymphoma HS-sultan cells, myeloma RPMI18266 cells [27], oral squamous carcinoma OSC-2 and OSC-4 cells [28], transformed human bronchial epithelial 21BES cells [29], and ovarian CAOV3 cancer cells [30], in curcumin-treated gingival fibroblasts and submandibular gland HSG carcinoma cells [31], in ovarian adenocarcinoma OVCAR-3 cervical carcinoma cells treated with ginkgetin [32], and in cactus pear extract-treated ovarian OVCA420 and SKOV3 cancer cells [33].

 Whereas DCHF-DA has been used to measure intracellular ROS, particularly H_2_O_2_, Nakazato et al. [27] used dihydroxyethidium (DHE) to measure intracellular O_2_^·−^ radicals. Upon uptake, cellular DHE is converted to ethidium, a fluorescent DNA intercalator, by cellular oxidants, particularly by O_2_^·−^. Elevated levels of intracellular O_2_^·−^ were detected in EGCG-treated lymphoma HS-sultan cells and myeloma RPMI18266 cells.

## 3. Strategies to Correlate Cytotoxic Effects with the Pro-Oxidant Nature of a Polyphenol

### 3.1. Intracellular Glutathione

Reduced glutathione (GSH), a thiol-containing tripeptide, is a significant contributor for maintaining the intracellular redox state and, as such, is an important component of the overall cellular defensive mechanisms against ROS. An important function of this intracellular antioxidant is to scavenge ROS produced during normal aerobic cellular respiration; if left unchecked, such ROS could oxidize and, thereby, damage nucleic acids, proteins, and lipids [5]. 

**Figure 3:**
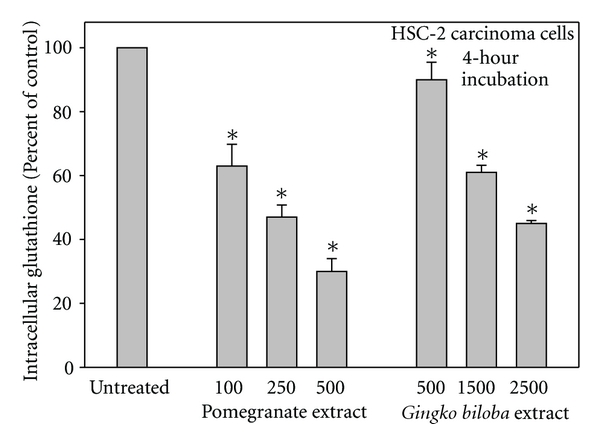
Decreases in intracellular reduced glutathione (GSH) following a 4-hour exposure of HSC-2 carcinoma cells in Dulbecco's modified Eagle medium amended with pomegranate extract [14] and with *Ginkgo biloba* extract [13]. The data are expressed as the arithmetic mean ± S.E.M.; in unexposed cells, the basal level was approximately 32 nmoles GSH/10^6^ cells.

 GSH directly interacts with H_2_O_2_ to yield oxidized glutathione (GSSG) and H_2_O. A hallmark indicator of oxidative stress is depletion of intracellular GSH (Figure 3), particularly in cancer cells which, when compared to normal cells, exhibit intrinsic oxidative stress, associated with increased metabolic activation, malfunctioning mitochondria, and oncogenic transformation [3]. Several studies have noted greater depletions of intracellular GSH in cancer, than in normal, cells upon their exposures to polyphenols, including green tea polyphenol extract [34], EGCG [12], black tea theaflavin mixture [35], theaflavin-3-gallate, theaflavin-3′-gallate [20], and TFdiG [19]. Normal cells maintain a proper intracellular redox status with their antioxidant enzymes and their sufficient supply of reduced GSH and thus are less susceptible to cytotoxic damage by pro-oxidant polyphenols [28, 30, 36]. 

 A strategy to correlate the pro-oxidant nature of a polyphenol with its cytotoxic potential is to cotreat cells with a polyphenol and a GSH depleter, as cells depleted of their intracellular GSH are hypersensitive to challenge with a pro-oxidant polyphenol. Such GSH depleters interrupt the glutathione redox cycle (GSH *↔* GSSG) by inhibiting those enzymes integral to the recycling processes. Commonly used enzyme inhibitors include 1,3-bis(2-chloroethyl)-*N*-nitrosourea (BCNU), DL-buthionine-[S,R]-sulfoximine (BSO), and 1-chloro-2,4-dichlorobenzene (CDNB). 

**Figure 4:**
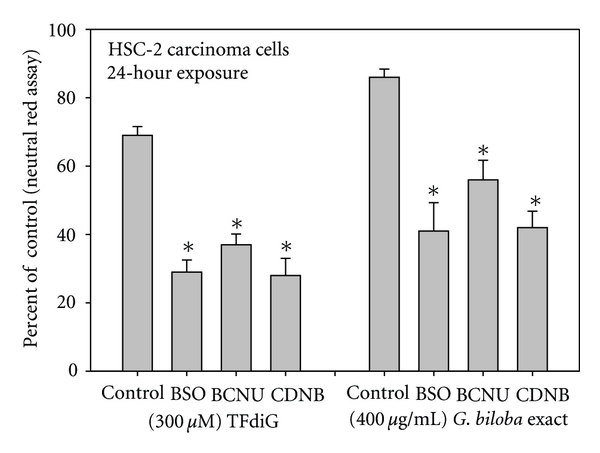
Comparative toxicities of theaflavin-3,3′-digallate (TFdiG) and *Ginkgo biloba* extract towards human oral HSC-2 carcinoma cells, untreated (control) and treated with glutathione depleters. Cell proliferation was quantified with the neutral red assay, a cell viability assay. Data are presented as the mean percent of control ± S.E.M. Data for the TFdiG study are from Schuck et al. [19] and for *G. biloba* from Babich et al. [13].

 BCNU, an irreversible inhibitor of glutathione reductase, inhibits the recycling of intracellular GSH thereby depleting intracellular stores of the tripeptide. Cotreatment of human oral cavity cells with BCNU potentiated the antiproliferative effects of protocatechuic acid [37]. GSH is synthesized enzymatically by a two-step process, involving *γ*-glutamylcysteine synthetase and glutathione synthetase. BSO, a selective inhibitor of *γ*-glutamylcysteine synthetase, prevents the resynthesis of GSH and thereby increases cellular sensitivity to oxidative stress. Treatment of human leukemic HL-60 cells with BSO potentiated oxidative DNA damage by EGCG [38]. The antiproliferative effects of eugenol [39], EGCG [12], theaflavin-3-gallate, theaflavin-3′-gallate [20], *G. biloba *extract [13], and gallic acid [40] were enhanced by cotreatment with BSO. Exposure to CDNB, which serves as a substrate and covalently binds to glutathione *S*-transferase, irreversibly depletes intracellular GSH. Potentiation of the antiproliferative effects of eugenol [39], EGCG [12], green tea polyphenol extract [34], *G. biloba *extract [13], and pomegranate extract [14] was noted upon pretreatment of cells with CDNB (Figure 4).

### 3.2. Lipid Peroxidation

Another classic indicator of oxidative stress is lipid peroxidation, a process whereby free radicals extract electrons from, and thereby damage, cell membranes. Such oxidative degradation progresses via a chain reaction, initiated by ROS interacting with polyunsaturated fatty acids to generate fatty acid radicals. In the presence of O_2_, a lipid peroxy fatty acid radical and thereafter a lipid peroxide radical form, with the chain reactions continuing until two free radicals interact to yield a nonradical species. Quantification of lipid peroxidation focuses on malondialdehyde (MDA), the end product of lipid peroxidation, and employs interactions between MDA and thiobarbituric acid (TBA), yielding thiobarbituric acid reactive substances (TBARSs), quantified by visible or fluorescence spectrophotometry. 

 Theaflavin-3-gallate and theaflavin-3′-gallate induced lipid peroxidation in human tongue CAL27 carcinoma cells [20]. Using rat hepatocyte cultures, Sahu et al. [41] demonstrated concentration-dependent lipid peroxidation upon exposure to nordihydroguaiaretic acid. Similarly, lipid peroxidation of freshly isolated lymphocytes was observed upon treatments with caffeic acid and gallic acid [42] and of erythrocytes by *G. biloba* extract [43]. Induction of lipid peroxidation by pro-oxidant polyphenols has been an underutilized assay.

### 3.3. ROS Scavenging Enzymes

Catalase enzymatically degrades H_2_O_2_ [2H_2_O_2_ → 2H_2_O + O_2_]. By far, the most common method to demonstrate that a polyphenol-induced cytotoxic response was due to the generation of H_2_O_2_ is to assess that adverse cellular response in the absence and presence of added catalase. The complete abolition of a cytotoxic effect of a polyphenol through cotreatment with catalase, apparently, would indicate that the adverse effect was mediated solely through the generation of H_2_O_2_. For example, the growth inhibitory and apoptotic-inducing effects of EGCG towards lymphoblastic leukemic Jurkat cells were completely suppressed by addition of catalase [23]. Coexposure of lymphoma HS-sultan cells to catalase completely prevented EGCG-induced apoptosis; downregulation of the antiapoptotic-associated proteins, Bcl-2 and Mcl-1, and of procaspase-3 after EGCG treatment was prevented by catalase pretreatment [27]. Yang et al. [25, 29] noted that the EGCG-induced apoptosis and elevated intracellular ROS in human colon H661 cancer cells were completely abolished by exogenously added catalase. 

**Figure 5:**
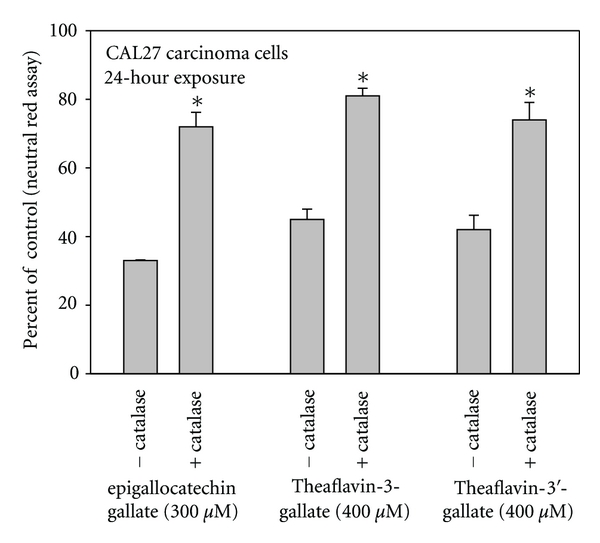
Comparative toxicities of epigallocatechin gallate (EGCG), theaflavin-3-digallate, and theaflavin-3′-gallate extract towards human oral HSC-2 carcinoma cells in the absence and presence of exogenously added catalase. Cell proliferation was quantified with the neutral red assay, a cell viability assay. Data are presented as the mean percent of control ± S.E.M. Data for EGCG are from Weisburg et al. [12] and for the black tea theaflavin monogallates are from Babich et al. [20].

 Many studies, however, showed that catalase afforded only a partial protection towards pro-oxidant polyphenol toxicities. Exposure of ovarian carcinoma OVCAR-3 to ginkgetin resulted in elevated intracellular ROS, growth inhibition, and apoptosis. Catalase afforded partial protection against growth inhibition and apoptosis, as noted by reductions in DNA fragmentation and double-stranded DNA breakage [32]. The antiproliferative effects of theaflavin-3-gallate, theaflavin-3′-gallate [20], TFdiG [19],* G. biloba *extract [13], and EGCG [12] to oral carcinoma cells were significantly lessened, but not completely abolished, in the presence of catalase (Figure 5). 

 In their studies on the response of human lung tumor H441 cells to EGCG, Yang et al. [25] noted that at similar EGCG concentrations, coexposures with exogenous catalase completely prevented apoptosis but only partially blocked the inhibition of cell proliferation. In subsequent studies with 21BES cells, Yang et al. [29] showed that exposures to TFdiG, EGCG, and EGC resulted in equivalent levels of intracellular H_2_O_2_ and in the induction of apoptosis. Yet, exogenously added catalase significantly prevented EGC- and EGCG-induced apoptosis but did not prevent TFdiG-induced apoptosis. Apparently, for these cells, EGCG and EGC induced apoptosis via oxidative stress, whereas TFdiG *per se* induced apoptosis by an ROS-independent mechanism. 

 EGCG-generated ROS in cell culture medium, and elevated levels of intracellular ROS were detected in human oral OSC-2 and OSC-4 EGCG-treated carcinoma cells. However, neither exogenous catalase nor *N*-acetylcysteine (NAC), an antioxidant, rescued the cells from the antiproliferative effects of EGCG [28]. Similarly, neither catalase nor NAC lessened the antiproliferative effect of (−)-catechin gallate to transformed oral S-G cells [18]. Apparently for these oral carcinoma cells, EGCG *per se *and catechin gallate *per se*, rather than their auto-oxidation ROS products, accounted for growth inhibition. However, Yamamoto et al. [28] observed that exogenous catalase partially rescued OCS-2 cells and more substantially rescued OSC-4 cells from EGCG-induced apoptotic cell death, as assayed by caspase-3 activation. Apparently, for the OSC-2 and OSC-4, the antiproliferative effects were due to EGCG *per se*, whereas the proapoptotic effects were due to EGCG-generated ROS.

 Lee et al. [22] studied the effects of gallic acid on gap-junction intracellular communication (GJIC), a process essential for maintaining homeostatic balance by modulating cell growth and differentiation and whose inhibition was linked to tumor promotion. GJIC in normal rat liver epithelial WB-344 cells was inhibited by the addition of authentic H_2_O_2_ and of gallic acid, a strong generator of H_2_O_2_. For H_2_O_2_, inhibition of GJIC was completely abolished by the addition of catalase, but for gallic acid, catalase only partially abolished this inhibition. Apparently, inhibition of GJIC by gallic acid was attributable both to the polyphenol *per se* and to H_2_O_2_.

 Cell culture medium amended with either green tea or with red wine inhibited proliferation of rat pheochromocytoma PC12 cells. Both amendments generated H_2_O_2_, and the addition of catalase completely abolished the antiproliferative effects of green tea, but only partially reduced that of red wine. Amendment with exogenous GSH had no significant effect on red wine toxicity. Apparently, H_2_O_2_ accounted for the total cytotoxic effect of green tea, but only partially for that of red wine [11]. It was suggested that the overall toxicity of red wine was a combination of H_2_O_2_ and of resveratrol, the main polyphenol in red wine, which has known antiproliferative effects [44].

 Apparently, the mediating effects of exogenous catalase on polyphenol cytotoxicity are varied. The complete abolition of a polyphenol-induced cytotoxic effect by exogenously added catalase indicates that the generated H_2_O_2_ alone was the toxic agent. Partial blockage of the polyphenol-induced cytotoxic effect by catalase indicates a dual mode of toxicity, the polyphenol *per se* in conjunction with its auto-oxidation ROS products. No lessening of cytotoxicity in the presence of catalase presumably is indicative of the polyphenol *per se* alone acting as the cytotoxic agent. However, other factors may have been involved, as the methodologies used in these studies may have had unforeseen effects on the biologic responses. For example, Vittal et al. [8] showed that cellular responses to EGCG included early responding genes whose expressions were H_2_O_2_ dependent and latter responding genes whose expressions were dependent upon EGCG *per se* (i.e., were H_2_O_2_ independent). The early responding genes were activated soon after exposure to EGCG, as early as 15 minutes afterexposure and with increased gene expression returning to basal levels by 3 to 6 hrs. A second cascade of gene expression followed between 3 and 10 hrs and a later response clustered between 12 and 36 hrs afterEGCG treatment. Thus, the time period of cell exposure to a polyphenol would be critical in evaluating the mediating influence of catalase. In many in vitro studies, the exposure period is 24 hr or less; in such a limited exposure period, attention would be focused towards H_2_O_2_-dependent genes, favoring their mediation by the addition of catalase. Another factor to consider is the pyruvate content of the cell culture medium, as it is a scavenger of H_2_O_2_. (The mediating influence of pyruvate is discussed in a later section). Finally, the toxicity of a polyphenol may be dependent upon ROS other than H_2_O_2_, in particular upon O_2_^·−^, whose activity is unaffected by catalase, but rather by SOD. As such, to rule out ROS as mediators of a polyphenol-induced cytotoxic effect, cell exposures to the polyphenol need to be done in the absence of antioxidant enzymes and in presence of catalase, of SOD, and of a combination of catalase and SOD. 

 As compared to catalase, fewer studies have been directed towards the effects of exogenous SOD on the toxic potency of pro-oxidant polyphenols. Superoxide radical is converted to H_2_O_2_, either spontaneously or enzymatically via SOD. Under physiological conditions, SOD, which requires metal cofactors, catalyzes the dismutation of the superoxide radical [2O_2_^·−^+ 2H^+^ → O_2_ + H_2_O_2_]. Elevation of intracellular O_2_^·−^ was observed in EGCG-treated lymphoma and myeloma cells [27]. Treatment of HL-60 leukemia cells with EGCG resulted in elevated intracellular ROS, with a concomitant increase in DNA damage, as detected in the alkaline comet assay. Coexposures either with SOD or with catalase largely prevented EGCG-mediated DNA damage and greatly reduced ROS formation; combinations of SOD and catalase completely inhibited intracellular ROS formation and DNA damage [45]. 

 The generation of O_2_^·−^ may be a major component of EGCG- [46] and of theaflavin-mediated toxicity, as Yen et al. [47] noted that DNA damage to EGCG-, EGC-, and theaflavin-treated Chang liver cells was associated with the generation of O_2_^·−^. In a cell-free system, SOD lessened the EGCG-mediated generation of H_2_O_2_ in sodium phosphate buffer (pH 7.8) and in RPMI medium, as well as suppressing the cytotoxicity of EGCG to Jurkat cells [23]. DNA breakage, as quantified in the comet assay, in human peripheral lymphocytes treated with resveratrol was inhibited by treatment with SOD [48].

### 3.4. Antioxidants

*N*-acetylcysteine (NAC), a derivative of the amino acid, L-cysteine with an acetyl group attached to the N atom, is a precursor in the synthesis of glutathione. NAC and GSH are typically used as exogenously added antioxidants to lessen the potency of pro-oxidant polyphenols. Using oral cancer cells as the bioindicators and inhibition of cell proliferation as the cytotoxicity endpoint, the potencies of EGCG [49] and of *G. biloba* extract [13] were lessened by NAC, of curcumin by NAC and GSH [50], and of green tea polyphenol extract by GSH [34]. Treatment of myeloid leukemic UF-1 cells with EGCG resulted in elevated intracellular ROS and in the induction of apoptosis, both of which were blocked upon coexposure with NAC [26]. Curcumin-induced onset of early apoptosis, as detected by the externalization of phosphatidylserine on cell surfaces of normal human gingival fibroblasts and human submandibular gland HSG carcinoma cells, and of the generation of intracellular H_2_O_2_, were lessened upon coexposures with exogenous NAC and GSH [31]. EGCG-induced activation of mitogen-activated protein kinases (MAPKs), release of cytochrome c, and apoptotic cell death of human colorectal HT-29 cells were significantly blocked in the presence of GSH and NAC. Interestingly, pretreatment with catalase failed to lessen MAPK activation and apoptotic cell death, indicating the involvement of ROS other than H_2_O_2_ [51].

### 3.5. Pyruvate

Pyruvate, amended to cell culture medium as sodium pyruvate, is a scavenger of H_2_O_2_; it nonenzymatically participates in a direct oxidative decarboxylation with H_2_O_2_ to yield acetate, carbon dioxide, and water [CH_3_COCOO^−^ + H_2_O_2 _→ CH_3_COO^−^ + CO_2_ + H_2_O] [52–54]. As such, pyruvate affords protection against pro-oxidant polyphenols. Exposure of human ovarian SKOV3 and CAOV3 cancer cells to EGCG resulted in growth inhibition, accompanied by increased intracellular levels of H_2_O_2_. Addition of pyruvate to the culture medium neutralized the cytotoxicity of EGCG [30]. 

 The antiproliferative toxicities of TFdiG [19], *G. biloba *extract [13], and pomegranate extract [14] to oral carcinoma HSC-2 cells were lessened in the presence of exogenous pyruvate. Studies with normal gingival fibroblasts also noted that pyruvate lessened the antiproliferative toxicity of caffeic acid, EGCG, TFdiG, black tea theaflavin mixture, and *G. biloba* extract [55]. Caspase-mediated cleavage of poly(ADP-ribose) polymerase (PARP), an indicator of apoptosis, occurred upon exposure of HSC-2 cells to 200 and 250 *μ*g/mL pomegranate extract. In cells cotreated with pomegranate extract and pyruvate, PARP cleavage was greatly reduced, indicating that pomegranate extract-induced apoptosis was a function of the induction of oxidative stress [14]. 

**Figure 6:**
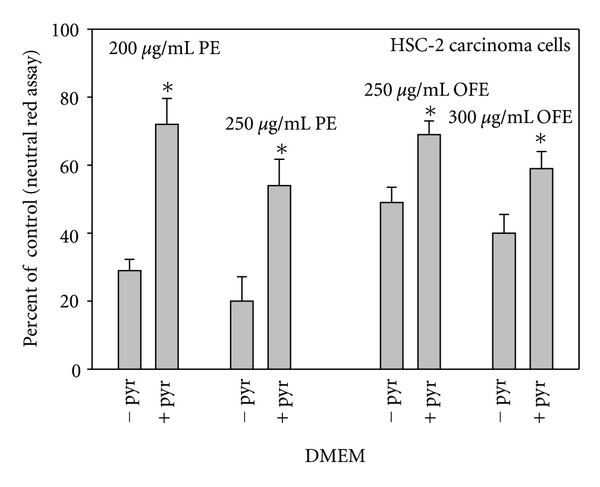
Comparative toxicities of pomegranate extract (PE) and olive fruit extract (OFE) towards human oral HSC-2 carcinoma cells, in Dulbecco's modified Eagle medium (DMEM) commercially formulated without pyruvate (−pyr) and with pyruvate (+pyr). Cell proliferation was quantified with the neutral red assay, a cell viability assay. Data are presented as the mean percent of control ± S.E.M. Data for pomegranate extract are from Weisburg et al. [14] and for olive fruit extract from Schuck (unpublished).

 Of the various mediators of pro-oxidant polyphenol cytotoxicity, pyruvate may have the most far reaching effects, as it is a component in some, but not in all, commercially available formulations of cell culture media. Furthermore, even within one particular type of medium, there may be different formulations, some with and some without pyruvate. Thus, RPMI, McCoy, Medium 199, and MEM typically lack pyruvate, whereas MEM*α* contains pyruvate. DMEM, the most commonly used cell culture medium, has various formulations, the majority of which contain pyruvate and others, much lesser in number, lack pyruvate.  Figure 6 shows the comparative cytotoxicities to HSC-2 cells of pomegranate extract and olive fruit extract in two commercially available formulations of DMEM, one with and the other without pyruvate. The mediating effect of pyruvate on H_2_O_2_ toxicity presumably is not well known, as the pyruvate content of the cell culture medium is seldom reported in the experimental design. Thus, cell responses to a pro-oxidant polyphenol may vary among laboratories, depending on the pyruvate content of the exposure media, which should be noted in publications. 

 In addition to directly scavenging exogenous H_2_O_2_, pyruvate has other properties to mediate oxidative stress. Pyruvate readily penetrates cells by a specific H^+^-monocarboxylic cotransporter [56] and is a readily oxidized fuel, enhancing the cytosolic energy state to maintain cellular functioning in the face of metabolic challenge [57]. Pyruvate dampens the mitochondrial generation of ROS, stabilizes mitochondrial ATP production compromised by oxidative stress, maintains the mitochondrial membrane potential under oxidative stress [58], and upregulates the expression of glutathione peroxidase, which is concerned with mitochondrial scavenging of H_2_O_2_ and in maintaining GSH levels [59]. The pyruvate content of the cell culture medium is an amendment that should be reckoned in studies of pro-oxidant polyphenols.

### 3.6. Cobalt

**Figure 7:**
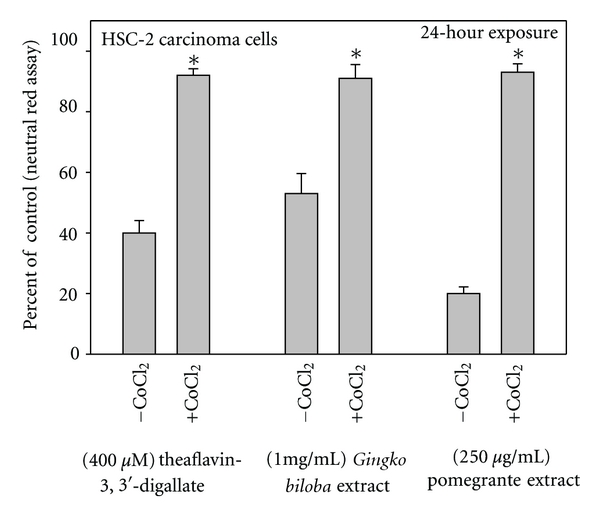
Comparative toxicities of theaflavin-3,3′-digallate, *Ginkgo biloba* extract, and pomegranate extract towards human oral HSC-2 carcinoma cells, untreated (control) and treated with cobalt chloride. Cell proliferation was quantified with the neutral red assay, a cell viability assay. Data are presented as the mean percent of control ± S.E.M. Data for the theaflavin-3,3′-gallate are from Schuck et al. [19], for *G. biloba* extract from Babich et al. [13], and for pomegranate extract from Weisburg et al. [14].

Divalent cobalt (Co^2+^) catalyzes the decomposition of H_2_O_2_ to H_2_O and O_2_, without an accompanying change in its valence [60]. Additions of mixtures of black tea polyphenols or of green tea polyphenols to cell culture medium generated significant amounts of H_2_O_2_, which progressively decreased in the presence of increasing concentrations of added Co^2+^, as CoCl_2_. As a scavenger of H_2_O_2_, amendments of cell culture medium with Co^2+^ afforded protection to transformed oral S-G cells from green and black tea polyphenol mixtures [61]. The antiproliferative effects of EGCG to oral HCS-2 and HSG carcinoma cells [49] and to normal gingival fibroblasts [62], of a black tea theaflavin mixture to transformed gingival GT1 fibroblasts and tongue carcinoma CAL27 cells [35], and of *Gingko biloba* extract [13] and of pomegranate extract [14] to HSC-2 cells were substantially lessened in the presence of CoCl_2_. Activation of caspase-3, an indicator of apoptosis, was noted in HSC-2 cells treated with pomegranate extract; when cotreated with pomegranate extract and CoCl_2_, activation of caspase-3 was greatly lessened [14]. In many of these studies [13, 14, 35], Co^2+^ afforded almost complete protection against polyphenol-induced growth inhibition (Figure 7). At the concentrations used in these studies, Co^2+^ usually provided greater protection from oxidative damage than catalase.

### 3.7. Iron

Nakagawa et al. [63] noted that *o*-phenanthroline, a Fe^2+^-chelating agent, suppressed EGCG-induced cell death in cultured osteoclastic cells. In a cell-free system, it was further shown that the reduction of Fe^3+^ to Fe^2+^ by EGCG triggered a Fenton reaction to form a highly reactive hydroxyl radical from the EGCG-generated H_2_O_2_ [H_2_O_2_ + Fe^2+^ → OH^·^ + OH^−^ + Fe^3+^]. Subsequent studies by Nakagawa et al. [23] noted that the growth inhibitory and apoptotic-inducing effects of EGCG towards Jurkat cells were partially suppressed by *o*-phenanthroline. These investigators suggested that the mode of cytotoxicity of EGCG was through the generation of H_2_O_2_, which triggered the Fe^2+^-dependent Fenton reaction, thereby generating highly toxic OH^·^ radicals to inhibit growth and to induce apoptotic cell death. 

 Lipid peroxidation in transformed oral S-G cells exposed to protocatechuic acid was enhanced in the presence of Fe^2+^ [37]. Similarly, Fe^2+^ potentiated the EGCG-lipid peroxidation towards oral cancerous and normal gingival fibroblasts [12]. In both studies, the polyphenol *per se* had little effect on lipid peroxidation, thus, indicating the involvement of a Fenton reaction.

### 3.8. Copper

Several studies have shown that plant polyphenols in the presence of metal ions cause oxidative damage to DNA. Divalent copper (Cu^2+^), one of the most redox sensitive metal ions in cells, is closely associated with chromatin. In a study by Bhat et al. [42], freshly isolated peripheral lymphocytes were treated with caffeic acid, and DNA damage was evaluated with the comet assay. DNA breakage, observed with 200 *μ*M caffeic acid, was progressively lessened in the presence of increasing concentrations of neocuproine, a Cu^1+^-specific chelating agent. Apparently, DNA breakage by caffeic acid involved endogenous copper, with Cu^1+^ an intermediate in the mechanistic pathway leading to DNA cleavage. Treatments with catalase and SOD reduced caffeic acid-mediated DNA damage; also, caffeic acid-mediated lipid peroxidation was lessened in the presence of neocuproine. Apparently, both Cu^1+^ and ROS were involved in oxidative damage by caffeic acid. To explain the mechanism for DNA damage by caffeic acid, it was postulated that the polyphenol bound both DNA and Cu^2+^ to form a ternary complex, leading to the reduction of Cu^2+^ to Cu^1+^, which subsequently interacted with polyphenol-generated H_2_O_2_ to afford OH^·^ radicals via a Fenton reaction. Similarly, damage of cellular DNA in lymphocytes was noted upon exposures to EGCG, gallic acid [42], and quercetin [48], with neocuproine sequestering DNA damage. Others have noted interactions between pro-oxidant polyphenols, ROS, and Cu^2+^→ Cu^1+^ to cause oxidative DNA damage although other mechanistic pathways, not involving a ternary complex but rather direct interactions between the polyphenol and Cu^2+^, were suggested [38, 64, 65]. 

## 4. Cellular Responses as Mediated by Pro-Oxidant Polyphenol Concentration

Three distinct cellular responses appear to result from exposure to polyphenols, with each response dependent upon the concentration and pro-oxidant nature of the polyphenols. (a) A mild exposure causes mild oxidative stress and thereby ignites cellular antioxidant defense systems. (b) An intermediate to high exposure gradually overwhelms the antioxidant defense systems and induces apoptotic cell death. (c) A very high exposure quickly overwhelms the cellular antioxidant defenses and causes oxidative damage leading to cell death by necrosis. In designing experiments to identify oxidative stress as the causative mechanism of cytotoxicity, a careful balance is needed between the generated ROS and the experimental variable (i.e., scavenger or potentiator of ROS toxicity) mediating oxidative stress. 

 As noted, glutathione, the major contributor for maintaining the redox state of the cell, exists in both a reduced (GSH) and an oxidized form (GSSG). Maintaining suitable levels of GSH is crucial to counteract oxidative stress and involves the transactivation of phase II detoxification/antioxidant genes encoding enzymes for GSH synthesis. Several gene response elements are involved in transcriptional regulation of GSH metabolism, including the antioxidant/electrophile response elements (AREs/EpREs), which have promoters with a specific consensus sequence of nucleotides that respond to molecules with *antioxidant* properties. Thus, plant-derived polyphenols, as antioxidants, regulate AREs/EpREs, leading to the synthesis of GSH. Although the specific mechanism is unclear, one approach suggested that the pro-oxidant nature of the polyphenol was the critical factor, in that auto-oxidation of the polyphenol generated ROS, which lessened the concentration of GSH, thereby to ignite transcriptional activation of *γ*-glutamylcysteine synthetase [5]. Exposure of COS-1 cells, an immortalized African green monkey kidney cell line, to noncytotoxic concentrations of quercetin enhanced synthesis of GSH through upregulation of *γ*-glutamylcysteine synthetase. Exposure of these cells to high concentrations of polyphenols led to elevated levels of ROS, which quickly depleted GSH stores and thereby increased cellular susceptibility to oxidative free radical attack, resulting in cell death by either apoptosis and/or necrosis [66]. 

 Studies with gingival fibroblasts showed that a 4-hour exposure to a nontoxic concentration of TFdiG [19] or of EGCG [12] stimulated the resynthesis of GSH, often to levels exceeding baseline. Early studies by Arrick et al. [67] noted that after a brief exposure to GSH depleters, cells rapidly resynthesized GSH, often overshooting normal levels. It has been suggested that a potential health benefit for the consumption of polyphenols at dietary levels is to generate low levels of ROS, so as to induce mild oxidative stress and thereby boost antioxidant defense systems to counteract potential challenge by elevated levels of ROS, perhaps through mitochondrial respiration [5, 9]. 

 Raza and John [68], using PC cells, derived from a pheochromocytoma of the rat adrenal medulla, showed that the level of EGCG was critical in evoking either a defensive mechanism or cell death by apoptosis. Low levels of EGCG (e.g., 50 *μ*M) apparently induced mild oxidative stress, igniting cellular antioxidant defenses, such as the stimulation of GSH synthesis and increased activity of glutathione-*S*-transferase. A higher level EGCG (e.g., 400 *μ*M) potentiated oxidative stress, as indicated by a persistent elevated intracellular level of ROS that overwhelmed and masked the antioxidant defensive mechanisms, leading to disruption of the intracellular GSH pool and an increase in lipid peroxidation. Also noted at 400 *μ*M EGCG was an increased expression of the enzyme, cytochrome P450 2E1, a member of the cytochrome P450 mixed-function oxidase system involved in the metabolism of ROS and a participant in cellular oxidative stress-related toxicity.

 In an interesting study by Hsuuw and Chan [69] with human breast cancer MCF-7 cells, responses were compared after exposures to moderate (20–50 *μ*M) and to high levels (100–400 *μ*M) of EGCG. Moderate levels of EGCG-induced apoptosis and elevated levels of EGCG induced cell necrosis (cell lysis). At moderate levels of EGCG, cell viability was lessened, and apoptosis was induced, and both correlated with increased oxidative stress, as indicated by intracellular generation of ROS, a loss of mitochondrial membrane potential, activation of caspase-3, caspase-9, and c-Jun-terminal kinase (JNK), and an increased expression level of Bax protein and a decreased level of Bcl-2 protein, thereby shifting the Bax-Bcl-2 ratio to favor apoptosis. Pretreatment of the MCF-7 cells with the antioxidants, NAC or *α*-tocopherol, attenuated intracellular ROS generation and rescued the cells from apoptotic death. Exposure to 25–50 *μ*M EGCG did not adversely affect ATP production; pretreatment with antimycin A, an inhibitor of mitochondrial respiratory activity, rescued the cells from apoptotic death, indicating that high levels of ATP were required to induce apoptosis. Conversely, at the high concentrations of EGCG, lesser levels of intracellular ROS and of apoptotic cells and only minor effects on caspase and JNK activations, on the Bax-Bcl-2 ratio, and on mitochondrial membrane potential were noted. Instead, cell death correlated with leakage of LDH, a sign of necrosis. Lowered ATP levels were observed at the high levels of EGCG. It was suggested that the switch from apoptotic death to necrosis was controlled by the intracellular level of ATP, with high ATP levels favoring apoptosis and decreased levels favoring necrosis. 

## 5. Studies on the Pro-Oxidant Effects of Polyphenols in Laboratory Animals and Humans

 The limited *in vitro* studies on the pro-oxidant nature of natural photochemical dwarfs the minuscule research with laboratory animal model systems. Li et al. [70] cited their study as “the first demonstration that EGCG induces ROS formation and consequently causes DNA oxidative damage in tumor cells in animals.” NCr nu/nu mice bearing human lung cancer H1299 xenograft tumors were maintained on a diet supplemented with 0.1, 0.3, and 0.5% EGCG. At the end of the 45-day experimental period, tumor growth was found to be dose dependently inhibited by EGCG. To correlate tumor inhibition with EGCG-induced oxidative stress, immunohistochemistry staining of the xenograft tumors was performed. The parameters evaluated included (a) the formation of the oxidative DNA-product, 8-hydroxyl-2′-deoxyguanosine (8-OHdG), a commonly used marker of oxidative stress, (b) the formation of phosphorylated histone 2A variant X (*γ*-H2AX), a cellular marker for the presence of double-strand DNA breaks, which can be caused by ROS, and (c) apoptotic activity, as measured by the induction of caspase-3. Dose-dependent induction of all three biochemical parameters was observed in the xenograft tumors.

 Animal studies using elevated doses of EGCG have reported hepatotoxicity linked to oxidative damage. Treatment of CF mice with a single high oral dose of 1,500 mg/kg EGCG or two once-daily doses of 750 mg/kg EGCG caused liver necrosis, associated with induction of apoptosis and increased lipid peroxidation and *γ*-histone 2AX protein expression. Plasma levels of 8-isoprostane, a nonenzymatic marker of arachidonic acid oxidation, were also increased upon EGCG treatment [71]. 

 Apparently, there are no other laboratory animal studies on the pro-oxidant effects of polyphenol nutraceuticals. 

 It has been noted that oral consumption of tea protected against carcinomas in the human oral cavity. Li et al. [72] showed that oral and topical administration of a mixed green tea preparation significantly reduced the size of oral precancerous lesions and the incidence of micronucleated oral mucosa cells in leukoplakia patients. Halder et al. [73] noted similar results in patients with oral leukoplakia who were administered a regimen of black tea. These studies did not evaluate whether the pro-oxidative nature of the teas was involved in chemoprevention. However, studies by Yang et al. [74], Lee et al. [75], and Lambert et al. [76] observed that holding tea solutions in the oral cavity or chewing tea leaves generated high salivary levels of catechins and theaflavins, accompanied with high levels of salivary H_2_O_2_. Catechin- and theaflavin-generated salivary H_2_O_2_ may have played a role in controlling the precancerous lesions in the studies by Li et al. [72] and Halder et al. [73].

## 6. Concluding Remarks

Cell response to a polyphenol challenge reflects a duality of toxicities, that of the polyphenol *per se* and that of the generated ROS, both of which modulate chemical signaling pathways leading to antiproliferative and apoptotic effects. To distinguish between the two chemical challenges, studies in the absence and in the presence of ROS scavengers are warranted. Some of the research strategies employed to elucidate oxidative stress are well established, for example, the use of glutathione depleters and ROS scavenging enzymes; other strategies are less known, that is, use of divalent cobalt and pyruvate to scavenge ROS. In studies in our laboratory, cobalt (usually, at 250 *μ*M CoCl_2_) was a more efficient scavenger of H_2_O_2_ than was catalase (usually, at 100 Units/mL). Most important for studies of oxidative stress is acknowledgement of the ROS scavenging property of pyruvate, as it is incorporated in formulations of some commercially available media and not in others. DMEM is probably the most utilized cell culture medium, yet its pyruvate is seldom noted in the experimental design. Of the numerous formulations of DMEM, relatively few lack pyruvate. As noted in our laboratory [55], the magnitude of the cellular response to a polyphenol differed significantly if the study was performed in DMEM without pyruvate as compared to using DMEM with pyruvate. As the pyruvate status of the medium is seldom incorporated into the description of the experimental design, it can be presumed that the ROS scavenging property of pyruvate is not well known.
